# Mass action models versus the Hill model: An analysis of tetrameric human thymidine kinase 1 positive cooperativity

**DOI:** 10.1186/1745-6150-4-49

**Published:** 2009-12-09

**Authors:** Tomas Radivoyevitch

**Affiliations:** 1 Department of Epidemiology and Biostatistics, Case Western Reserve University, Cleveland, Ohio 44106, USA

## Abstract

**Background:**

The Hill coefficient characterizes the extent to which an enzyme exhibits positive or negative cooperativity, but it provides no information regarding the mechanism of cooperativity. In contrast, models based on the equilibrium concept of mass action can suggest mechanisms of cooperativity, but there are often many such models and often many with too many parameters.

**Results:**

Mass action models of tetrameric human thymidine kinase 1 (TK1) activity data were formed as pairs of plausible hypotheses that per site activities and binary dissociation constants are equal within contiguous stretches of the number of substrates bound. Of these, six 3-parameter models were fitted to 5 different datasets. Akaike's Information Criterion was then used to form model probability weighted averages. The literature average of the 5 model averages was K = (0.85, 0.69, 0.65, 0.51) μM and k = (3.3, 3.9, 4.1, 4.1) sec^-1 ^where K and k are per-site binary dissociation constants and activities indexed by the number of substrates bound to the tetrameric enzyme.

**Conclusion:**

The TK1 model presented supports both K and k positive cooperativity. Three-parameter mass action models can and should replace the 3-parameter Hill model.

**Reviewers:**

This article was reviewed by Philip Hahnfeldt, Fangping Mu *(nominated by William Hlavacek) *and Rainer Sachs.

## Background

The Hill model [1] characterizes cooperativity with a single number, but it cannot discriminate cooperativity mediated by enzyme activity changes versus substrate binding affinity changes. In contrast, models based on the equilibrium concept of mass action (Eqs. 2-4 below) accomplish this, but to be used, methods that deal with multiple models and models that are over-parameterized [2] need to be developed.

This paper yields a literature model of tetrameric human thymidine kinase 1 (TK1) activity data [3-7] that could be used in network models of dNTP supply [8]. TK1 is important because it rate-limits the absorption of thymidine and analogs such as the cancer imaging marker 3'-^18 ^F-fluoro-3'-deoxy-fluorothymidine (FLT) [9,10].

## Results

### Hill Analyses of TK1 Data

**Figure 1:**
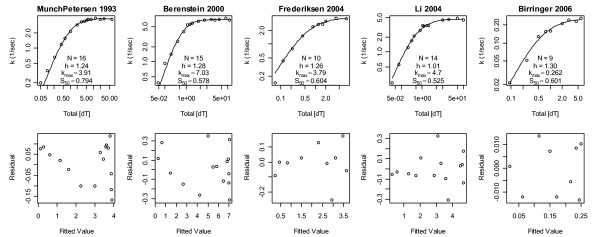
**Non-weighted nonlinear least squares Hill model fits to 5 datasets**. Residuals of Munch-Petersen *et al*. 1993 and Berenstein *et al*. 2000 show a trend from positive to negative values across lower fitted values, i.e. poor fits. The dataset of Birringer *et al*. 2006 is an outlier in that its *k*_max _is 15-30-fold smaller than those of the other datasets. The data of Li *et al*. 2004 is different in that its Hill coefficient is ~1 rather than 1.24-1.30. The 2004 data show that the variance increases with increases in fitted values, as it should since activities cannot be negative and thus the activity variances must decrease with decreasing expected values. The Hill coefficients presented here approximately equal those of the original publications.

The empirical Hill model of the average activity of an enzyme per catalytic site as a function of the total substrate concentration [*S*_T_] is(1)

where *k*_max _is the maximum activity obtained in the limit of high/saturating substrate concentrations, *S*_50 _is the total substrate concentration at *k *= 1/2*k*_max_, and if the Hill coefficient *h *is greater than 1 or less than 1 the enzyme is said to exhibit positive or negative Hill cooperativity, respectively. Non-weighted nonlinear least squares fits of this model to five human tetrameric TK1 datasets [3-7] are shown in Fig. 1. Collectively, these fits suggest a literature median TK1 Hill model of *k*_max _= 4/sec, *S*_50 _= 0.6 μM and *h *= 1.25; hereafter, all units are in μM and seconds.

**Figure 2:**
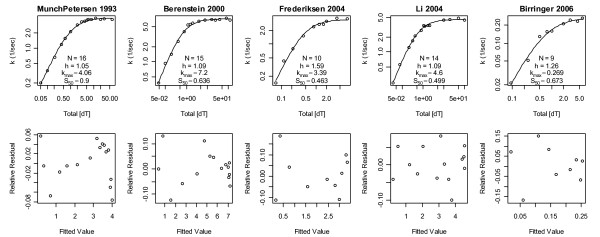
**Weighted nonlinear least squares Hill model fits**. Reciprocal data squared weights of 1/k^2 ^were used to minimize the sum of squared *relative *residuals. Compared to Fig. 1 the Hill coefficients here strike a better balance between low and high substrate concentrations. The Hill coefficient of Li *et al*. 2004 is now similar to those of the 1993 and 2000 datasets. The Hill coefficient of Frederiksen *et al*. 2004 is now an outlier at *h *= 1.6.

The Hill model has an amplitude scale parameter *k*_max_, a concentration scale parameter *S*_50_, and thus only one shape parameter *h*. It therefore cannot represent enzymes that require different shape parameters in the regions [S_T_] >*S*_50_ versus [S_T_] <*S*_50_. Further, if non-weighted least squares is used and the data are not transformed to stabilize the variance, *k *measured closer to saturating concentrations will be over weighted because fluxes must be positive and their variance must therefore decrease as flux measurements approach zero. Thus, if the variance is not stabilized, and/or weights are not used, *h *will adjust itself more to fit curvature at [S_T_] >*S*_50 _than at [S_T_] <*S*_50_. That this is a problem in Fig. 1 is apparent from the correlations in the residuals of the first two datasets. These residuals clearly indicate a poor fit at low substrate concentrations, as one would expect if data in this region were not given adequate weight in the sum of squared errors. To correct this, squared error weights of 1/*k*^2 ^were used to increase the importance of deviations at smaller *k *values; here *k *denotes data and *k *(e.g. in the Hill model) is the expected value of this data (both symbols will be used to denote both collections of points and individual points, and in rare cases where statements are true only for the *j*th data point, these symbols will be replaced by *k*_(j) _and *k*_(j)_, respectively). The results are shown in Fig. 2. The relative residuals therein are more homogeneous and less trendy than the absolute residuals in Fig. 1. Differences in *h *values between these figures suggest that TK1 average activity shapes may indeed differ between the regions [S_T_] >*S*_50 _and [S_T_] <*S*_50_. Figure 2 also suggests that the literature median *h *should be 1.1 rather than 1.25. Further, it shows that the third dataset now stands alone with *h *= 1.6; as removal of this dataset's lowest concentration data point lowers this *h *to an acceptable value of 1.28, this data point will be excluded from subsequent analyses.

Figures 1 and 2 strongly suggest that the literature collectively favors positive cooperativity over no (and negative) cooperativity, since *h *≤ 1 was never observed and the probability of 10 coin tosses of the same sign in a row is 2*2^-10 ^= 1/512. Based on this literature wide conclusion, the Michaelis-Menten model will be removed from the space of plausible mass action models below, i.e. it will not be fitted to the data and thus will not contribute to model averages.

### Mass Action Based Models

A model of tetrameric human thymidine kinase 1 in quasi-equilibrium with its substrate thymidine is given by the following total concentration constraints (TCCs):(2)

where [E_T_] and [S_T_] are the total enzyme *tetramer *and substrate concentrations (these are the manipulated experimental design variables, or system inputs) and implicit in Eq. (2) are the following mass action equilibrium equations (which should also be viewed as definitions of the *complete *dissociation constants used):(3)

Equation (2) is a coupled system of polynomials in the free concentrations [E] and [S]. It is solved numerically in the R package Combinatorially Complex Equilibrium Model Selection (ccems) [11] by embedding it into a parent system of ordinary differential equations (ODEs) which solves the polynomials at steady state [12]. The free concentrations so obtained are then back substituted into Eq. (3) to estimate the enzyme-substrate complex concentrations [ES^i^] and these are then substituted into(4)

to form the expected activity *k*. Here, the *k*_*i *_are per site average activities of enzyme tetramers that have *i *occupied substrate sites, averaged over the occupied sites, and *k*, on the other hand, is the expected measured activity as an average over all enzyme catalytic sites, whether they are occupied by substrate or not. Equations (2-4) comprise what is called the full model because it is fully parameterized, i.e. as of yet, no constraints have been placed on any of its 8 parameters. The TCCs above are also called the system equations and Eq. (4) is also called the output linkage [12]. Thus, this is a two-stage model where *K *are system parameters, *k*_*i *_are output linkage parameters, and *k*_(j) _= *k*_(j) _+ ε_j _where <ε_j_> = 0 and the variance σ^2^(ε_j_) depends on the fitted value *k*_(j)_; ε_j _is measurement noise and <ε_j_> is its mean.

If the concentration of free substrate [S] approximately equals [S_T_] because the maximum [E_T_] in the data is much less than the minimum positive [S_T_], as is the case in the five datasets analyzed here [3-7], ODE computations needed to solve the TCCs in Eq. (2) can be avoided because the second TCC then reduces to [S] = [S_T_] and by substitution, the first TCC then reduces to(5)

where Eq. (3) is now(6)

If Eqs. (6) and Eq. (5) are substituted into Eq. (4), the net result is the following 8-parameter rational polynomial model that replaces Eqs. (2-4):(7)

Here, Eqs. (2-4) and Eq. (7) are both mass action based full models, but in contrast to Eqs. (2-4), the result in Eq. (7) is independent of [E_T_], i.e. the approximation [S] = [S_T_] made above is associated with fluxes *v *= 4*k *[E_T_] scaling linearly in [E_T_].

Though Eq. (7) is valid without any approximation if [S_T_] is replaced by [S], free substrate concentrations are often unknown unless [S] ≈ [S_T_], and in these cases it is best to state the approximation explicitly in the model as a reminder of its presence, for although Eq. (7) holds under the conditions ([E_T_] < 0.1 nM) of the data analyzed [3-7], it does not hold when [E_T_] is in the range of [S_T_]. Note that *k*_*i *_and *K *estimated using Eq. (7) with low [E_T_] data (where [S] ≈ [S_T_]) still apply to Eqs. (2-4) with Eq. (2) solved using ODEs, but solved using ODEs, the model is also valid at high [E_T_] where [S] < [S_T_].

**Figure 3:**
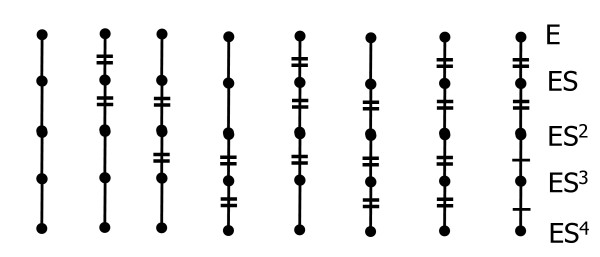
***K *equality hypotheses**. Edges are binary dissociation constants and nodes are complexes shown on the right. Edges marked = or -- are alleged equal. Unmarked edges are independently estimated. An analogous figure arises for complex specific activities *k*_*i *_if edges are mapped to the nodes below them.

To generate *K *equality hypotheses, the complete dissociation constants in Eqs (2-7) must be rewritten as products of per-site binary dissociation constants:

where specific binary reactions are indicated by underscores in the subscripts. It is these binary dissociation constants(8)

that can plausibly equal each other. Such binary *K *equality hypotheses are restricted here to contiguous blocks shown in Fig. 3 on grounds that if one ligand disrupts a protein structure, it is unlikely that an additional ligand will return it to one of its previous forms, i.e. it is unlikely that an additional ligand will return a model parameter to one of its previous values. This argument applies analogously to specific enzyme complex activities *k*_*i *_(see Fig. 3 legend).

The 8 binary *K *models in Fig. 3 were automatically generated and paired with each of 8 analogous *k *models to form a product space of 64 models. The hypothesis

which corresponds to the Michaelis-Menten model

was then excluded from the model space based on the Hill analysis conclusion of Figs. 1 and 2 that some TK1 positive cooperativity must exist. The resulting 63 models were then fitted to the five datasets using nonlinear least squares; the Box-Cox transformation [13] with λ = 0.5

was used to stabilized the variance. The Akaike Information Criterion (AIC) was then computed for each model: for normal errors and small sample sizes, AIC = 2*P + 2*P(*P*+1)/(*N*-*P*-1) + N*log (2π) + N*log (SSE/*N*) + *N *where *P *is the number of estimated parameters (including the variance), *N *is the number of data points, and SSE is the sum of squared errors [2]. The AICs were then used to form model probabilities e^ΔAIC^/Σe^ΔAIC ^where ΔAIC is the difference between a model's AIC and the minimum of all model AICs [2]. The model probabilities were then used to form model probability weighted averages of the parameters. To minimize the influence of low probability over-parameterized models whose parameter estimates had escaped to large values, averages were formed as exponentials of model probability weighted averages of logarithms of the parameter estimates (for *K *= *e*^Δ*G*/*RT *^this corresponds to forming averages of Gibbs free energy changes).

**Figure 4:**
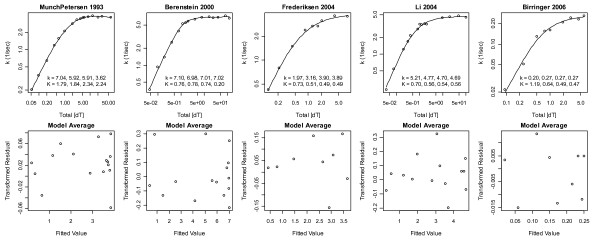
**Model averages**. A Box-Cox transformation with λ = 0.5 was used to stabilize the variance. The residuals shown are thus transformed. For parameter estimate interpretations see text.

Using the vector notation **K **≡ () μM and **k **= (*k*_1_, *k*_2_, *k*_3_, *k*_4_) sec^-1^, the model averages formed using all 63 of the 3- to 8-parameter models (Fig. 4) suggested the following mechanisms: the 1^st ^dataset, with **K **= (1.8, 1.8, 2.3, 2.2) and **k **= (7, 6, 6, 3.6), supports **K **negative cooperativity (which maps to Hill coefficients *h *< 1) annihilated by stronger **k **negative cooperativity (which, counterintuitively, maps to *h *> 1, see below); the 2^nd ^dataset, with **K **= (.76, .78, .74, .20), supports enhanced 4^th ^substrate binding; the 3^rd ^dataset supports enhanced activity and affinity of complexes with 2 or more bound substrates; the 4^th ^dataset supports **K **positive cooperativity combined with **k **negative cooperativity; and the 5^th ^dataset supports both **K **and **k **positive cooperativity (coefficients are given in Fig. 4).

**Figure 5:**
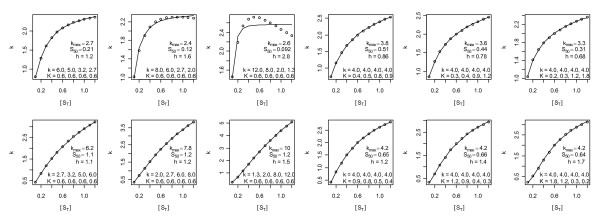
**Hill model fits to simulated data**. In the first three columns **K **= (0.6, 0.6, 0.6, 0.6) was held fixed and the spread of **k **values was increased to simulate greater degrees of **k **negative (top row) and positive (bottom row) cooperativity. Analogously, in the 4^th ^to 6^th ^columns, **k **= (4, 4, 4, 4) was held fixed and **K **was varied. These simulations demonstrate that increases in **k **negative cooperativity map to increases in Hill positive cooperativity until a point is reached (e.g. in the 2^nd ^column) where the fit is too poor to accept. Meanwhile, **k **positive cooperativity in the bottom row of columns 1 to 3 and **K **cooperativity in columns 4 to 6 map to Hill coefficients *h *in an expected manner.

To characterize the relationship between Hill cooperativity and **k **and **K **cooperativity, the Hill model was fitted to samples of various simulated mass action models. The results (Fig. 5) show that **k **negative cooperativity maps to *h *> 1, though with poorer fits as the cooperativity becomes stronger. Meanwhile, **k **positive cooperativity, and **K **positive or negative cooperativity, map to *h *in expected ways. These results suggest that **K **and **k **work together to create *h *> 1 in the 4^th ^dataset and that, for the 1^st ^dataset, **k **negative cooperativity (which creates Hill positive cooperativity) annihilates slight **K **negative cooperativity (which creates slight Hill negative cooperativity).

**Figure 6:**
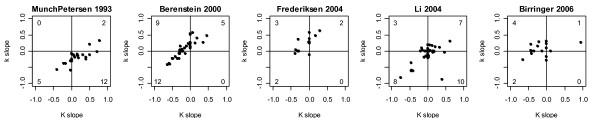
**Parameter trend distributions**. The **k **and **K **of models with probabilities >10^-6 ^were normalized by their means and fitted to straight lines versus the integers 1 to 4 to yield normalized slopes, i.e. parameter trends. The number of models within each quadrant is shown in the plots; models on axes (constant **k **and/or constant **K**) are excluded from these counts. Based on these counts, the 2^nd^, 3^rd^, and 5^th ^datasets group together in that none of them have a model in the lower right quadrant.

To obtain single measures of trends, the **K **and **k **of models that had model probabilities >10^-6 ^were normalized by their means and fitted to straight lines versus the integers 1 to 4. The two slopes obtained in this way are shown as points in Fig. 6. This figure shows that the 2^nd^, 3^rd^, and 5^th ^datasets form a group in that they have no models in the lower right quadrant and more models in the left two quadrants than in the right two quadrants, i.e. consistent with **k **and **K **working together to implement Hill positive cooperativity.

### Literature Model

To provide one mathematical representation of the TK1 literature for use in network models of dNTP supply [8], an average of the models in Fig. 4 was formed. To give **k **values of the 5^th ^dataset fair representation, **k **means were averaged independent of **k **shapes (which were averaged as percentages of means). This yielded the model **K **= (1.0, 0.9, 0.9, 0.8) and **k **= (4.0, 4.3, 4.4, 4.1).

The percent contributions of 3- and 4-parameter models to the model averages, indexed by the 5 datasets, were (.04, 90, 100, 80, 100) and (97, 10, 0, 20, 0), respectively, i.e. the 1^st ^dataset requires 4-parameter models and the 3^rd ^and 5^th ^datasets (with lowest sample sizes) require only 3-parameter models. If the three highest [S_T_] data points of the 1^st ^dataset are deleted to eliminate a post *k*_max _downturn in *k *at high [S_T_] (Fig. 4), 97% of the 1^st ^dataset's model average is then due to 3-parameter models. Since, if deleted, the 1^st ^dataset's model average would have been **K **= (1, 1, 1, 0.9) and **k **= (4, 4, 4.5, 4.1), i.e. with **K **positive (instead of negative) cooperativity that is consistent with the other datasets, and since, if deleted, the slopes of the 1^st ^dataset in Fig. 6 then move into the upper left quadrant to yield a plot similar to those of the 2^nd^, 3^rd ^and 5^th ^datasets, these 3 data points were excluded from all subsequent analyses.

**Figure 7:**
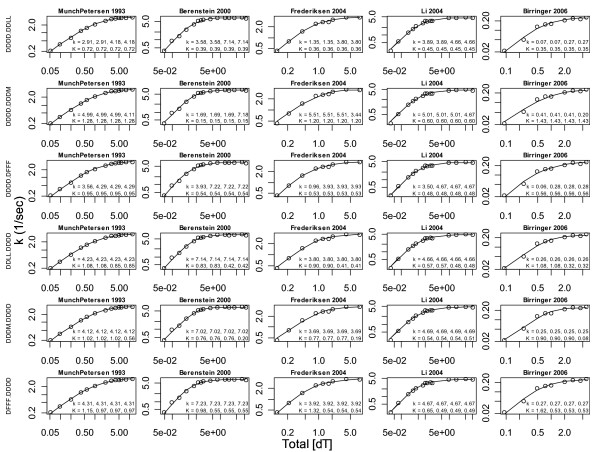
**The 3-parameter mass action models fit the data well**. The bottom three rows support **K **positive cooperativity, the 1^st ^and 3^rd ^support **k **positive cooperativity, and the 2^nd ^row supports **k **negative cooperativity in all but the second dataset.

Reasons to restrict the model space to the six 3-parameter models DFFF.DDDD, DDLL.DDDD, DDDM.DDDD, DDDD.DFFF, DDDD.DDLL and DDDD.DDDM (here **K **components are on the left, **k **components are on the right, and letters are the same when parameters that correspond to their positions equal each other) include:

1. All 6 of these models fit all 5 datasets well (Fig. 7), as one might expect since *h *not far from 1 implies that the data are not far from the Michaelis-Menten model that lives within each of these models if two parameters equal each, i.e. it is reasonable to expect that each model can adjust its 3^rd ^parameter to meet differences between *h *= 1 and *h *= 1.1 to 1.3.

2. Some 4-parameter models fitted to their own simulated data in the absence of noise across physiological thymidine levels of 0.1 μM to 1.2 μM [14] showed signs of over-parameterization (i.e. failure to return true parameter values and sensitivity to initial parameter values).

3. The 4-parameter model contribution to the 4^th ^dataset was mostly due to DFFF.DFFF which is already represented in the model average via the two 3-parameter models DFFF.DDDD (32%) and DDDD.DFFFF (25%), but the 4-parameter model claims an unrealistic *k*_1 _of 25, i.e. it is likely over-parameterized and causing an undue impact on the average; other models with similar issues are also eliminated if only 3-parameter models are fitted.

**Figure 8:**
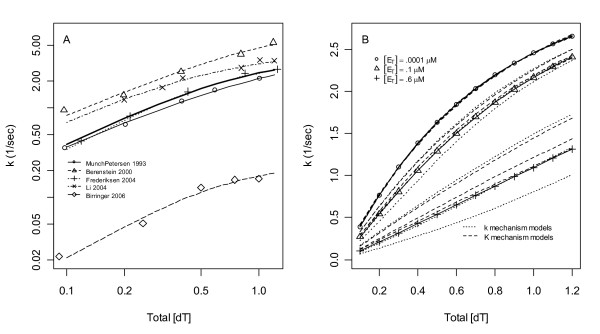
**Literature model average**. **A) **Model averages of Table 2 were equally weighted to form the literature average in Eq. (9) (thick line). **B) **The literature average was extrapolated to [E_T_] in the range of [S_T_]. Although solutions to Eqs. (2-4) match their rational polynomial approximation in Eq. (7) at [E_T_] = 0.0001 μM (o), this approximation fails at [E_T_] = 0.1 μM (Δ) and fails drastically at [E_T_] = 0.6 μM (+). All six of the 3-parameter mass action models (dashed lines) fit the literature average at [E_T_] = 0.0001 μM (o) but diverge in their extrapolated predictions at [E_T_] = 0.1 μM and [E_T_] = 0.6 μM.

The model space was thus restricted to 3-parameter models and a total of 4 outliers were removed (recall that the lowest [S_T_] data point of the 3^rd ^dataset was removed based on the Hill analysis of Fig. 2). The net results of these actions are that now the 1^st ^dataset favors a **k **mechanism with both **k **positive and **k **negative cooperativity, the 2^nd ^dataset fully favors **K **positive cooperativity, the 3^rd ^and 4^th ^datasets support balances of **k **and **K **mechanisms, and the 5^th ^dataset favors **K **positive cooperativity, see Table 1. These statements are reflected in the dataset model averages in Table 2 (Fig. 8A) and in literature averages of the 3-parameter models in Table 3. The average of the averages in Table 2 is(9)

**Table 1:** Models that contributed more than 5% to a model average.

Dataset	Model	Weight	K_1_	K_2_	K_3_	K_4_	k_1_	k_2_	k_3_	k_4_
1	DDDD.DDDM	0.444	1.28	1.28	1.28	1.28	4.99	4.99	4.99	4.11

1	DDDD.DDLL	0.298	0.72	0.72	0.72	0.72	2.91	2.91	4.18	4.18

1	DDDM.DDDD	0.219	1.02	1.02	1.02	0.56	4.12	4.12	4.12	4.12

2	DDDM.DDDD	0.978	0.76	0.76	0.76	0.20	7.02	7.02	7.02	7.02

3	DFFF.DDDD	0.393	1.32	0.54	0.54	0.54	3.92	3.92	3.92	3.92

3	DDDD.DFFF	0.335	0.53	0.53	0.53	0.53	0.96	3.93	3.93	3.93

3	DDDD.DDLL	0.204	0.36	0.36	0.36	0.36	1.35	1.35	3.80	3.80

3	DDLL.DDDD	0.059	0.90	0.90	0.41	0.41	3.80	3.80	3.80	3.80

4	DFFF.DDDD	0.400	0.65	0.49	0.49	0.49	4.67	4.67	4.67	4.67

4	DDDD.DFFF	0.314	0.48	0.48	0.48	0.48	3.50	4.67	4.67	4.67

4	DDLL.DDDD	0.118	0.57	0.57	0.48	0.48	4.66	4.66	4.66	4.66

4	DDDD.DDDM	0.074	0.60	0.60	0.60	0.60	5.01	5.01	5.01	4.67

5	DFFF.DDDD	0.545	1.62	0.53	0.53	0.53	0.27	0.27	0.27	0.27

5	DDLL.DDDD	0.234	1.08	1.08	0.32	0.32	0.26	0.26	0.26	0.26

5	DDDD.DFFF	0.192	0.56	0.56	0.56	0.56	0.06	0.28	0.28	0.28

**Table 2:** Model averages of the datasets

Dataset	K_1_	K_2_	K_3_	K_4_	k_1_	k_2_	k_3_	k_4_
1	1.02	1.02	1.01	0.89	4.0	4.05	4.51	4.14

2	0.76	0.76	0.75	0.20	7.0	6.99	7.01	7.02

3	0.73	0.51	0.49	0.49	2.0	3.16	3.90	3.89

4	0.56	0.50	0.49	0.49	4.3	4.65	4.69	4.67

5	1.19	0.64	0.49	0.47	0.2	0.27	0.27	0.27

**Table 3:** Literature averages of the 3-parameter models

Model	Weight^a^	K_1_	K_2_	K_3_	K_4_	k_1_	k_2_	k_3_	k_4_
DFFF.DDDD	1.34	1.14	0.62	0.62	0.62	4.08	4.08	4.08	4.08

DDLL.DDDD	0.47	0.89	0.89	0.50	0.50	4.02	4.02	4.02	4.02

DDDM.DDDD	1.26	0.80	0.80	0.80	0.31	3.95	3.95	3.95	3.95

DDDD.DFFF	0.84	0.61	0.61	0.61	0.61	2.08	4.18	4.18	4.18

DDDD.DDLL	0.56	0.82	0.82	0.82	0.82	2.78	2.78	3.70	3.70

DDDD.DDDM	0.53	0.93	0.93	0.93	0.93	3.52	3.52	3.52	3.92

^a^The total weight equals 5 datasets.

(thick curve in Fig. 8A). If a single predictive model of TK1 rates is needed in a model of dNTP supply [8], use of Eq. (9) is recommended. If a single model is to be fitted to TK1 data, Fig. 7 suggests that any of the 3-parameter mass action models can be used instead of the Hill model and Table 3 suggests that DFFF.DDDD and DDDM.DDDD should perhaps be preferred.

### Extrapolations

If the literature model in Eq. (9) is simulated at [E_T_] = 0.1 nM and sampled at 12 [S_T_] points between 0.1 μM to 1.2 μM, these "data" (Fig. 8B circles) are fitted well by a Hill model with *k*_max _= 4.18/sec, *S*_50 _= 0.714 μM and *h *= 1.14. This Hill model is independent of [E_T_] and thus does not deviate from the circles in Fig 8B as [E_T_] increases into the range of [S_T_]. In contrast, with [E_T_] in activated lymphocytes estimated to be 0.04 μM based on TK1 tetramers of 100 kDa and an enzyme concentration of 4 μg/ml [4], in some cells [E_T_] could reach 0.1 μM, and at this concentration, and much more so if [E_T_] reached 0.6 μM, drastic differences in the shape of the response are obtained if Eq. (2) is solved exactly using ODEs [12]. The differences between the circles and triangles and circles and plus signs in Fig. 8B are the errors that would result if the fitted Hill model were used at [E_T_] = 0.1 μM or 0.6 μM, respectively. Meanwhile, the six 3-parameter mass action models also provide excellent fits to the [E_T_] = 0.1 nM simulated data, but they change shapes and thus extrapolate better to [E_T_] = 0.1 μM and [E_T_] = 0.6 μM (dashed lines Fig. 8B). If the 3-parameter mass action models are capable of representing TK1, experiments at [E_T_] = 0.6 μM should yield a *k *response that lies within the range of curves spanned by these models in Fig. 8B; if such *k *data falls below the literature average (plus signs in Fig. 8B), support will be gained for a **k **mechanism since only one 3-parameter model lies below the average and it is a **k **model, and if the data falls slightly above the literature average support will be gained for a **K **mechanism. In all of these extrapolations it is assumed that mass action equilibriums of Eqs. (3) are rapid relative to changes in [S_T_], i.e. that Eqs. (2-4) can be coupled to -d [S_T_]/dt = 4*k*([S_T_], [E_T_]) [E_T_] to form a differential algebraic equation (DAE) model of TK1.

## Discussion

**Figure 9:**
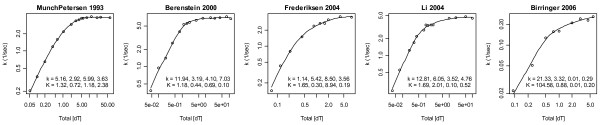
**Full model fits**. Noise is captured by the parameter estimates of the 8-parameter full model much more than by the expected values of its response. See text.

**Figure 10:**
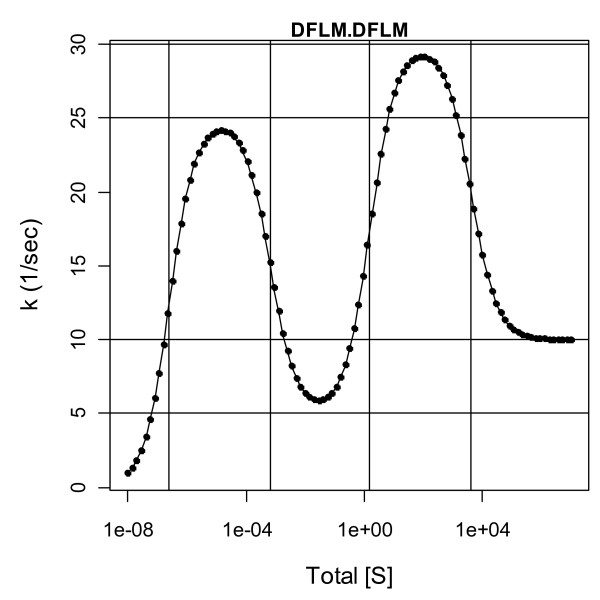
**The model K = (10^-6^, 10^-3^, 1, 10^3^) and k = (100, 10, 40, 10)**. Horizontal lines are *k*_1_/4, 2*k*_2_/4, 3*k*_3_/4 and 4*k*_4_/4 and vertical lines are concentrations of [S_T_] at which half the species are [ES^i^] and the other half are [ES^i+1^]; from Eq. (8) the vertical lines are at *K*_1_/4, 2*K*_2_/3, 3*K*_3_/2 and 4*K*_4_.

The 8-parameter full model fits the datasets without capturing much noise in its predictions (Fig. 9) and this is consistent with unrealistically different parameter values being needed to create a wavy response in Fig. 10. Thus, for this model space, over-parameterization manifests itself as highly correlated parameters (to a point of becoming non-identifiable) rather than over fits of expected values (e.g. as in the case of *n*-th order polynomial perfect fits to *n*+1 data points). The problem that arises when models have essentially non-identifiable parameters is that optimizations can then escape to large and meaningless parameter values. Though low model probabilities typically annihilate the influence of such models on model averages, with many models fitted, some will have parameter estimates that are large enough to cause noise in the overall model average parameter estimates. Such models are of little to no value if the goal is to carry information to a lower scale of mechanisms, though they are perhaps still useful as predictors of reaction rates (i.e. when information is being carried to higher scales of metabolic networks). By using a basis set of only 3-parameter models, monotonic parameter estimate trends resulted (Eq. 9). As monotonic trends are more biologically plausible than noisy trends, this suggests that the parameter estimates absorbed relatively little noise, i.e. that restriction to a parsimonious model basis set of only 3-parameter models kept noise out of the model average parameter estimates.

In Fig. 10 horizontal lines are shown at *k*_1_/4, 2*k*_2_/4, 3*k*_3_/4 and 4*k*_4_/4 and vertical lines are shown at *K*_1_/4, 2*K*_2_/3, 3*K*_3_/2 and 4*K*_4_. As a model approaches the 2-parameter limit of the Michaelis-Menten model, the horizontal lines become evenly spaced and the vertical lines position themselves at *K*_m_/4, 2*K*_m_/3, 3*K*_m_/2, and 4*K*_m_. In this limit plateaus and peaks disappear and only two parameters can be estimated accurately regardless of the density, range, precision and accuracy of the measurements. As deviations from this limit arise, a third parameter can be identified, and with greater changes more parameters can be estimated. If an enzyme's profile has no apparent peaks or plateaus on its rise up, it may never yield more than 3 or 4 meaningful parameter estimates. And if measurements are restricted to lie within a grid of physiologically relevant concentrations, the number of parameters that can be estimated can only be less; rationale for such restrictions is that if two models do not differ over any physiologically relevant reactant concentrations, either can be used.

It is known that TK1 is tetrameric at the physiologic ATP levels (2.5 to 3 mM) of the TK1 data analyzed [4,15,16]. The literature model provided by Eq. (9) should thus be valid when applied to such situations. If predictions are needed for situations where TK1 dimers and tetramers coexist, two models may be needed, one for the dimer population and one for the tetramer population. Such situations may exist when TK1 is phosphorylated on serine 13 [6].

When the number of catalytic sites is greater than the number of substrates, as in the proposed experiments with [E_T_] = 0.6 μM (and thus [TK1_T_] = 2.4 μM), most catalytic sites will process at most 1 or 2 substrates across the time course of product formation. With average conversion times of 0.25 seconds once a substrate is bound to a catalytic site, assuming exponentially distributed processing times, the probability that a particular bound substrate has not been converted to product within one second is e^-4 ^= 0.018. Thus, if the substrates are all initially bound, less than 2% of [S_T_] will remain after 1 second. Note that if no enzyme has more than one substrate bound during the time course of the measurements, at most *k*_1 _and *K*_1 _can be estimated from the data. Indeed, differences in *k*_1 _dominate the 3-parameter model separations at [E_T_] = 0.6 μM in Fig. 8B where, in the limit of low [S_T_], the number of tetramers with one substrate approaches [S_T_] and the rate law thus approaches 1/4 *k*_1 _[S_T_].

## Conclusion

All six of the 3-parameter mass action models have two advantages over the Hill model (which also has 3 parameters): 1) they provide a means of extrapolation to [E_T_] in the range of [S_T_], and 2) conditional on their truth, they yield more interesting parameter estimates. Though the Hill model was useful in that it indicated that the mutual Michaelis-Menten submodel could be excluded from the space of mass action models, the advantages of mass action models, and averages thereof (Eq. 9), suggest that they are better final end products of enzymological research.

## Methods

### Data

All of the datasets were digitized using plotDigitizer [17].

### Analysis

The R package ccems was used to generate and fit the models [11].

## Competing interests

The author declares that he has no competing interests.

## Authors' contributions

TR performed the work and wrote the paper.

## Reviewer's comments

### Reviewer's report 1

#### Philip Hahnfeldt, Tufts University

1. Please provide an example of how TCC models are used to generate product formation time courses wherein [S_T_] decreases. 2. In your 2008 paper you included model conjectures that certain complete dissociation constants were approximately infinite. Why were these not explored here? 3. Perhaps it should be emphasized that if a tetramer has *j *catalytic sites occupied by substrates, the average activity parameter *k*_j _may not be representative of the activities of the individual sites. Finally, 4. it would be helpful if it was explicitly stated how data in reciprocal seconds used in this paper were obtained from data provided in other units in the cited papers.

### Radivoyevitch's Responses

**Figure 11:**
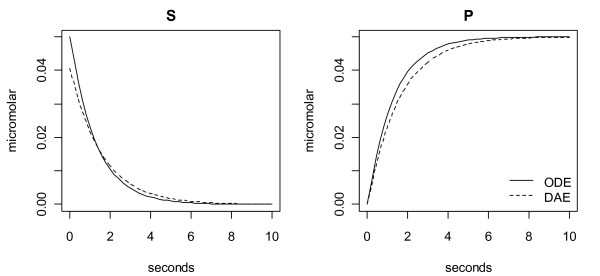
**Rational polynomial versus TCC product formation time courses**. The literature average model of Eq. (9) [i.e. **K **= (0.85, 0.69, 0.65, 0.51) and **k **= (3.3, 3.9, 4.1, 4.1)] was simulated for [S_T_] = 0.05 μM and [E_T_] = 0.05 μM using the rational polynomial model of Eq. (7; solid curve) and TCCs of Eqs. (2-4; dotted).

**Figure 12:**
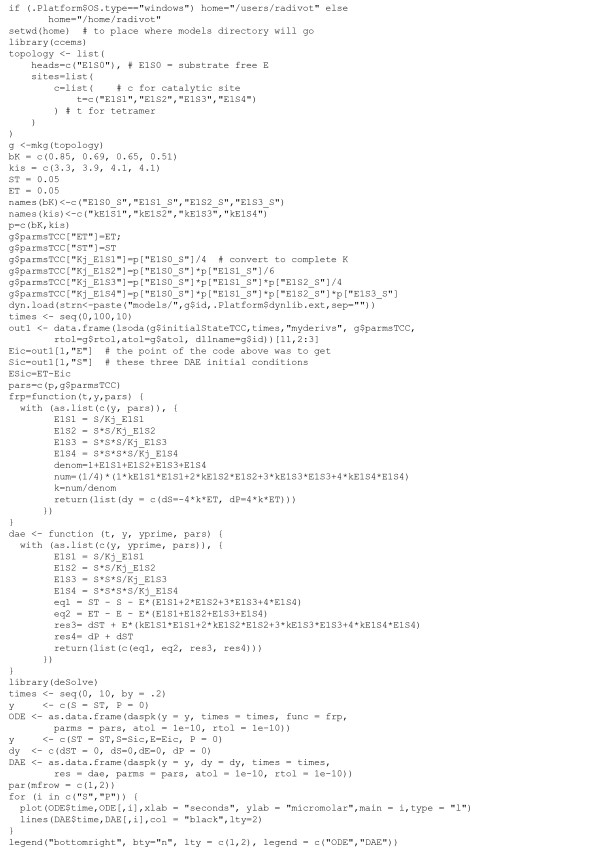
**R codes used to generate Figure 11**. The top half of these codes find the initial free concentrations [E] and [S] that are then used by the differential algebraic equation (DAE) solver daspk of the R package deSolve.

1) Suppose [S_T_] = 0.05 μM, [E_T_] = 0.05 μM and that the literature model in Eq. (9) holds. Fig. 11 shows product formation time courses generated using ODEs using the rational polynomial model of Eq. (7) (solid curve) as well as DAEs using the TCC model of Eqs. (2-4) (dotted curve). For details, R codes used are provided in Fig. 12.

2) Model averaging makes more sense for binary K models than for complete K models since K infinity hypotheses are difficult to average. Though the use of association rather than dissociation constants may appear to remedy this, if one considers ΔG to be the true underlying parameter, the choice then is really between positive versus negative infinity, rather than zero and infinity, and the problem persists. Thus, to keep the paper focused on model averaging, I decided not to consider K infinity hypotheses.

3) Using activity averages was necessary because only one polynomial term [E][S]^j ^in the equations represents tetramers with *j *occupied catalytic sites, so there is no way to distinguish site activities. Indeed, the *j*th bound substrate could have no activity and this could either decrease the average, if the other site activities stayed the same, or increase it, if the other site activities increase enough to offset the loss.

4) If R and ccems are installed, load(ccems) followed by ?TK1 yields Table 4. In this table E, S = dT, and X = ATP are total concentrations in μM, and the product flux measurements v are in μmol/min per mg of enzyme in datasets 1 through 4 and in pmoles/min in the 5^th ^dataset. Since TK1 is 25 kDa = 25 mg/μmole, 1 mg of TK1 equals 0.04 μmoles of enzyme and the conversion between v and k in 1/seconds is thus k = v/(.04*60). For the 5^th ^dataset the concentration of the enzyme is 306 pM and the reaction vessel is 30 μL, so the total amount of enzyme is 30 μL * (0.000306 pmoles/μL) = 0.00918 pmoles and thus k = v/(0.00918*60).

**Table 4:** TK1 literature data

E^a^	S	X	V	Figure	Year	First Author	set	k
8.00E-05	0.051474	2500	0.489	4	1993	MunchPetersen	1	0.20375

8.00E-05	0.097841	2500	0.861	4	1993	MunchPetersen	1	0.35875

8.00E-05	0.201282	2500	1.57	4	1993	MunchPetersen	1	0.654167

8.00E-05	0.39749	2500	2.85	4	1993	MunchPetersen	1	1.1875

8.00E-05	0.589425	2500	3.79	4	1993	MunchPetersen	1	1.579167

8.00E-05	1.005871	2500	5.14	4	1993	MunchPetersen	1	2.141667

8.00E-05	2.026393	2500	6.91	4	1993	MunchPetersen	1	2.879167

8.00E-05	3.045455	2500	8.04	4	1993	MunchPetersen	1	3.35

8.00E-05	4.033816	2500	8.35	4	1993	MunchPetersen	1	3.479167

8.00E-05	5.081395	2500	8.74	4	1993	MunchPetersen	1	3.641667

8.00E-05	5.933333	2500	8.9	4	1993	MunchPetersen	1	3.708333

8.00E-05	8.107143	2500	9.08	4	1993	MunchPetersen	1	3.783333

8.00E-05	12.37845	2500	9.42	4	1993	MunchPetersen	1	3.925

8.00E-05	19.91091	2500	8.94	4	1993	MunchPetersen	1	3.725

8.00E-05	27.90274	2500	9.18	4	1993	MunchPetersen	1	3.825

8.00E-05	64.38849	2500	8.95	4	1993	MunchPetersen	1	3.729167

2.00E-04	0.04535	2500	0.907	2a	2000	Berenstein	2	0.377917

2.00E-04	0.096983	2500	2.25	2a	2000	Berenstein	2	0.9375

2.00E-04	0.197633	2500	3.34	2a	2000	Berenstein	2	1.391667

2.00E-04	0.394156	2500	6.07	2a	2000	Berenstein	2	2.529167

2.00E-04	0.802521	2500	9.55	2a	2000	Berenstein	2	3.979167

2.00E-04	1.183486	2500	12.9	2a	2000	Berenstein	2	5.375

2.00E-04	1.587112	2500	13.3	2a	2000	Berenstein	2	5.541667

2.00E-04	2.008547	2500	14.1	2a	2000	Berenstein	2	5.875

2.00E-04	4.047619	2500	15.3	2a	2000	Berenstein	2	6.375

2.00E-04	7.819905	2500	16.5	2a	2000	Berenstein	2	6.875

2.00E-04	16.1	2500	16.9	2a	2000	Berenstein	2	7.041667

2.00E-04	24	2500	16.4	2a	2000	Berenstein	2	6.833333

2.00E-04	40	2500	16.7	2a	2000	Berenstein	2	6.958333

2.00E-04	79.9	2500	17.6	2a	2000	Berenstein	2	7.333333

2.00E-04	120	2500	16.1	2a	2000	Berenstein	2	6.708333

2.00E-04	0.0699	2500	0.342	4a	2004	Frederiksen	3	0.1425

2.00E-04	0.119	2500	1.03	4a	2004	Frederiksen	3	0.429167

2.00E-04	0.214	2500	1.92	4a	2004	Frederiksen	3	0.8

2.00E-04	0.427	2500	3.63	4a	2004	Frederiksen	3	1.5125

2.00E-04	0.846	2500	5.81	4a	2004	Frederiksen	3	2.420833

2.00E-04	1.24	2500	6.46	4a	2004	Frederiksen	3	2.691667

2.00E-04	1.65	2500	6.49	4a	2004	Frederiksen	3	2.704167

2.00E-04	2.04	2500	7.55	4a	2004	Frederiksen	3	3.145833

2.00E-04	4.05	2500	8.76	4a	2004	Frederiksen	3	3.65

2.00E-04	8.01	2500	8.62	4a	2004	Frederiksen	3	3.591667

2.00E-04	0.075862	2500	1.32	2a	2004	Li	4	0.55

2.00E-04	0.035823	2500	0.566	2a	2004	Li	4	0.235833

2.00E-04	0.198658	2500	2.96	2a	2004	Li	4	1.233333

2.00E-04	0.314729	2500	4.06	2a	2004	Li	4	1.691667

2.00E-04	0.402344	2500	5.15	2a	2004	Li	4	2.145833

2.00E-04	0.806024	2500	6.69	2a	2004	Li	4	2.7875

2.00E-04	1.007353	2500	8.22	2a	2004	Li	4	3.425

2.00E-04	1.202703	2500	8.01	2a	2004	Li	4	3.3375

2.00E-04	1.638507	2500	8.34	2a	2004	Li	4	3.475

2.00E-04	2.01467	2500	8.24	2a	2004	Li	4	3.433333

2.00E-04	9.72	2500	10.8	2a	2004	Li	4	4.5

2.00E-04	19.7	2500	11.1	2a	2004	Li	4	4.625

2.00E-04	49.7	2500	11.6	2a	2004	Li	4	4.833333

2.00E-04	100	2500	10.9	2a	2004	Li	4	4.541667

0.000306	0.0917	6000	0.012	4a	2006	Birringer	5	0.021786

0.000306	0.249	6000	0.0282	4a	2006	Birringer	5	0.051198

0.000306	0.498	6000	0.0709	4a	2006	Birringer	5	0.128722

0.000306	0.747	6000	0.0862	4a	2006	Birringer	5	0.1565

0.000306	0.996	6000	0.0883	4a	2006	Birringer	5	0.160312

0.000306	1.99	6000	0.116	4a	2006	Birringer	5	0.210603

0.000306	3	6000	0.133	4a	2006	Birringer	5	0.241467

0.000306	4.49	6000	0.127	4a	2006	Birringer	5	0.230574

0.000306	5.99	6000	0.143	4a	2006	Birringer	5	0.259622

^a^E = TK1, S = dT and X = ATP are in total concentrations in μM. Product dTMP fluxes v are in umol/min per mg of enzyme in datasets 1-4 and in pmoles/min in the 5^th ^dataset.

### Reviewer's report 2

#### Fangping Mu, Los Alamos National Laboratory (nominated by Bill Hlavacek, LANL)

In this report, the author analyzes the cooperativity of tetrameric human thymidine kinase 1 (TK1) activity. Literature data suggests that activity is marked by positive cooperativity rather than no cooperativity or negative cooperativity. The author formulates mass-action models to study possible mechanisms of cooperativity. Five literature data sets with 16, 15, 10, 14 and 9 data points were collected. The data sets were used to estimate the values of eight parameters in the mass-action models via a fitting procedure. The author finds that the best-fit mass-action models are marked by positive cooperativity.

The mass-action models have eight parameters that are adjusted to fit only 9 to 16 data points. The small number of data points may not be sufficient to identify the parameters in the models considered. The author uses AICs to measure the quality of model selection, but multiple models seem to fit the data equally well. The statistics are estimated from the training data, and it is not known how well the models can be used for testing. In other words, the models may not be predictive.

Positive cooperativity is supported by Hill coefficient fitting to the data sets. Without a 3D structure analysis of protein-ligand binding, a pure statistical fitting procedure may not provide much insight into the mechanism of cooperativity.

#### Radivoyevitch's Responses

Regarding your first point, only fits of the six 3-parameter models were used to produce the final model, i.e. what appeared to be an 8-parameter model was thus the model probability weighted model average of 6 fitted 3-parameter models. I agree that as shown in Fig. 7, each of the six 3-parameter models fits each of the datasets well, but I disagree that the models may not be predictive. Indeed, the whole point of Fig. 9 is to state that even the fitted 8-parameter model is predictive, i.e. there is very little high frequency noise in the expected values generated by these models. Instead of yielding poor predictions, because this model space has a fairly constrained range space, as demonstrated by the extremely different K values needed to introduce oscillations in Fig. 10, here over-parameterized models lead to noisy model parameter estimates as shown in Fig. 9, i.e. rather than poor prediction, the problem here is that we have weak parameter estimate inferences. If interests are in a TK1 model that will be inserted into a higher scale model of dNTP supply, such over-parameterization may not be a major concern. On the other hand, if the goal is to use the model to reach lower scale enzyme structures, stronger parameter estimate inferences are desirable. The basis set of six 3-parameter models yields monotonic parameter value trends in the literature average model average and this suggests that the parameter estimates are not noisy. Note too that TK1 structural information, namely that it is a tetramer (by size exclusion) at the ATP concentrations of the experiments, was used to constrain the model space to the forms explored. The goal then is to have the model capture as much information as possible, and I believe the analysis presented comes closer to this than any previous TK1 data analysis.

### Reviewer's report 3

#### Rainer K. Sachs, University of California at Berkeley

No comment.

## References

[B1] Hill AV The possible effects of the aggregation of the molecules of hemoglobin on its dissociation curves J Physiology 1910 40

[B2] Burnham KP Anderson DR Model Selection and Multimodel Inference: A Practical-Theoretic Approach 2002 Springer-Verlag

[B3] Berenstein D Christensen JF Kristensen T Hofbauer R Munch-Petersen B Valine, not methionine, is amino acid 106 in human cytosolic thymidine kinase (TK1). Impact on oligomerization, stability, and kinetic properties J Biol Chem 2000 275 41 32187 32192 10.1074/jbc.M005325200 10924519

[B4] Munch-Petersen B Tyrsted G Cloos L Reversible ATP-dependent transition between two forms of human cytosolic thymidine kinase with different enzymatic properties J Biol Chem 1993 268 21 15621 15625 8340387

[B5] Birringer MS Perozzo R Kut E Stillhart C Surber W Scapozza L Folkers G High-level expression and purification of human thymidine kinase 1: quaternary structure, stability, and kinetics Protein Expr Purif 2006 47 2 506 515 10.1016/j.pep.2006.01.001 16473525

[B6] Li CL Lu CY Ke PY Chang ZF Perturbation of ATP-induced tetramerization of human cytosolic thymidine kinase by substitution of serine-13 with aspartic acid at the mitotic phosphorylation site Biochem Biophys Res Commun 2004 313 3 587 593 10.1016/j.bbrc.2003.11.147 14697231

[B7] Frederiksen H Berenstein D Munch-Petersen B Effect of valine 106 on structure-function relation of cytosolic human thymidine kinase. Kinetic properties and oligomerization pattern of nine substitution mutants of V106 Eur J Biochem 2004 271 11 2248 2256 10.1111/j.1432-1033.2004.04166.x 15153115

[B8] Bradshaw PC Samuels DC A computational model of mitochondrial deoxynucleotide metabolism and DNA replication Am J Physiol Cell Physiol 2005 288 5 C989 1002 10.1152/ajpcell.00530.2004 15634740

[B9] von Forstner C Egberts JH Ammerpohl O Niedzielska D Buchert R Mikecz P Schumacher U Peldschus K Adam G Pilarsky C Grutzmann R Kalthoff H Henze E Brenner W Gene expression patterns and tumor uptake of 18F-FDG, 18F-FLT, and 18F-FEC in PET/MRI of an orthotopic mouse xenotransplantation model of pancreatic cancer J Nucl Med 2008 49 8 1362 1370 10.2967/jnumed.107.050021 18632830

[B10] Atkinson DM Clarke MJ Mladek AC Carlson BL Trump DP Jacobson MS Kemp BJ Lowe VJ Sarkaria JN Using fluorodeoxythymidine to monitor anti-EGFR inhibitor therapy in squamous cell carcinoma xenografts Head Neck 2008 30 6 790 799 10.1002/hed.20770 18286491 PMC3942889

[B11] Combinatorially Complex Equilibrium Model Selection http://epbi-radivot.cwru.edu/ccems/overview.html

[B12] Radivoyevitch T Equilibrium model selection: dTTP induced R1 dimerization BMC Syst Biol 2008 2 1 15 10.1186/1752-0509-2-15 18248678 PMC2268910

[B13] Box GE Cox DR An Analysis of Transformations Journal of the Royal Statistical Society Series B 1964 26 2 211 252

[B14] Holden L Hoffbrand AV Tattersall MH Thymidine concentrations in human sera: variations in patients with leukaemia and megaloblastic anaemia Eur J Cancer 1980 16 1 115 121 7358072 10.1016/0014-2964(80)90116-4

[B15] Sherley JL Kelly TJ Human cytosolic thymidine kinase. Purification and physical characterization of the enzyme from HeLa cells J Biol Chem 1988 263 1 375 382 3335503

[B16] Munch-Petersen B Cloos L Tyrsted G Eriksson S Diverging substrate specificity of pure human thymidine kinases 1 and 2 against antiviral dideoxynucleosides J Biol Chem 1991 266 14 9032 9038 2026611

[B17] Plot Digitizer http://plotdigitizer.sourceforge.net/

